# Extraperitoneal Spillage in Ruptured Tubo-Ovarian Abscess: A Case Report and Review of Literature

**DOI:** 10.7759/cureus.63324

**Published:** 2024-06-27

**Authors:** Sunita Murmu, Mamta Datta, Rajdeep Kour, Karan Agarwal

**Affiliations:** 1 Obstetrics and Gynaecology, Tata Main Hospital, Jamshedpur, IND; 2 Obstetrics and Gynaecology, Manipal Tata Medical College, Jamshedpur, IND; 3 Surgery, Manipal Tata Medical College, Jamshedpur, IND

**Keywords:** anterior abdominal wall, sepsis, pelvic inflammatory diseases, extra-peritoneal spillage, ruptured tubo-ovarian abscess

## Abstract

We report herein a case of a 43-year-old female with a ruptured tubo-ovarian abscess complicated by sepsis and extraperitoneal spillage into the anterior abdominal wall. The patient initially presented with acute abdominal pain and septic shock. Pelvic computed tomography revealed a collection in the abdomen that suggested a ruptured tubo-ovarian abscess, which dissects into the right rectus plane. There was a complete resolution of sepsis following surgical drainage. The patient underwent a hysterectomy with a bilateral salpingo-oophrectomy.

## Introduction

A tubo-ovarian abscess is a severe complication arising from inadequately treated or untreated acute pelvic inflammatory disease, characterized by an inflammatory mass that predominantly affects the fallopian tube, ovary, and surrounding pelvic organs. A ruptured abscess can give rise to severe sepsis and warrant emergency surgery [1]. Whenever rupture occurs, the content is usually accumulated in the peritoneal cavity. A rupture of a tubo-ovarian abscess with extra-peritoneal spillage is a rare event. We report an unusual case of ruptured tubo-ovarian abscess with extra-peritoneal spillage into the rectus sheath plane.

## Case presentation

A 43-year-old sexually active woman, para 1, live 0, abortion 1, with a significant history of heavy menstrual bleeding, presented to the emergency room in a state of shock and severe abdominal pain. The pain was widespread, particularly in the right iliac and lumbar regions, associated with anorexia and vomiting, and aggravated on movements towards the right side. She had a low-grade fever for a week. She reported experiencing chronic abdominal pain for a month, which gradually increased. She had undergone appendicectomy and laparoscopic myomectomy twice in the past. The patient was ill and tachypneic upon admission. Her vital signs were as follows: body temperature: 100.4 °F; pulse rate: 130 beats per minute; blood pressure: 88/56 mm Hg. The patient’s physical and abdominal examination revealed guarding and tenderness in the lower abdomen and right lumbar region. On palpation, a huge, firm mass with restricted mobility was arising from the pelvis, occupying the lower abdomen and extending up to the umbilicus. Multiple laboratory tests were performed, including a complete blood count, c-reactive protein, serum procalcitonin, and tumour markers, which yielded the following results (Table 1).

**Table 1:** Laboratory values of patient at the time of admission. CEA: carcinoembryonic antigen; AFP: alpha-fetoprotein.

Laboratory parameters	Patient’s value	Reference range
Hemoglobin	5.3 g/dl	11.5–16.5 g/dl
White blood cells	29,430 per mm^3^	4000–11,000 per mm^3^
Platelets	488,000 per mm^3^	150,000–410,000 per mm^3^
C-reactive protein	19.38 mg/dl	0.08–0.79 mg/dl
Procalcitonin	1.52 ng/ml	0.02–0.3 ng/ml
CA-125	188.8 U/ml	<35 U/ml
CA 19.9	41.7 U/ml	<37 U/ml
CEA	0.92 ng/ml	<3 ng/ml
AFP	12.85 ng/ml	<8.5 ng/ml
Beta HCG	2.21 mIU/ml	<5 mIU/ml

**Figure 1:**
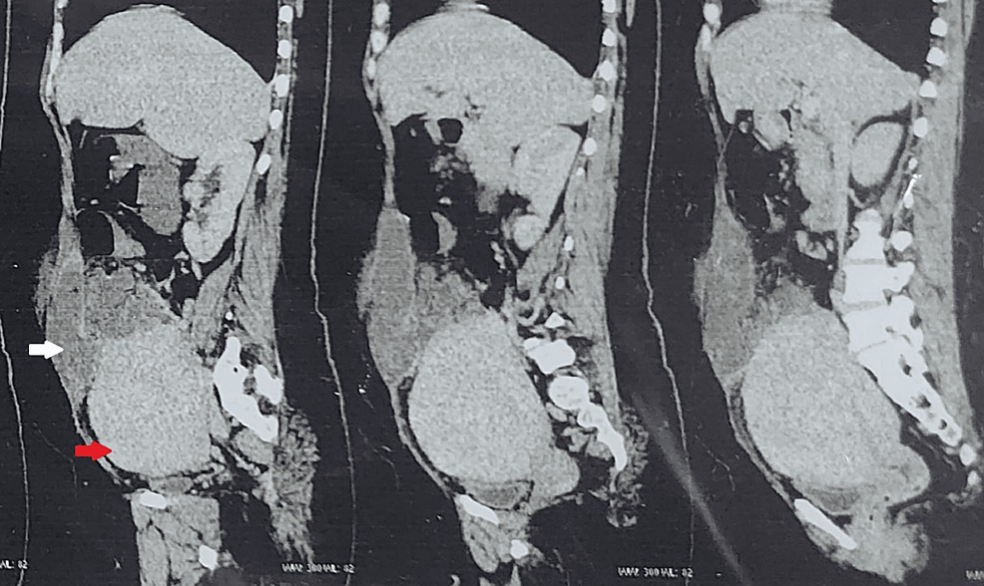
Sagittal planes of computed tomography showing ruptured tubo-ovarian abscess dissecting into rectus sheath plane (white arrow) and grossly bulky uterus (red arrow).

The computed tomography of the abdomen and pelvis revealed hypodense collection in the right adnexa extending to the right iliac fossa and dissecting into the right rectus sheath plane of the anterior abdominal wall and a grossly bulky uterus (Figure 1).

**Figure 2:**
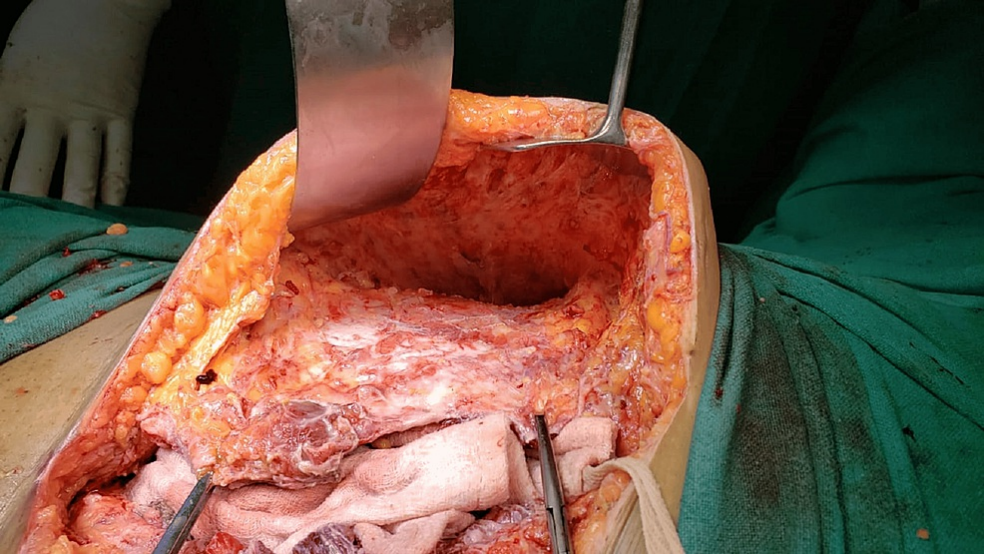
Anterior abdominal wall after drainage of pus and debridement of subcutaneous fat.

An emergency laparotomy was performed based on the clinical diagnosis of septic shock. We drained about 1000 ml of foul-smelling pus. A ruptured tubo-ovarian abscess, including the right ovary and tube, covered with a small intestine, was revealed. The pelvic anatomy was grossly distorted as a result of previous surgeries. Pus pockets were present between the uterus and right adnexa and had an extraperitoneal extension to the right rectus plane and subcutaneous fat of the anterior abdominal wall. The uterus was enlarged due to a big fibroid and was adhered anteriorly to the bladder and abdominal wall. The patient underwent a total abdominal hysterectomy with a bilateral salpingo-oophrectomy. The abscess extending to the anterior abdominal wall was drained, and debridement of subcutaneous fat was done above the external oblique muscle (Figure 2). The abdominal wall and peritoneal cavity were copiously irrigated, and closed suction drains were placed intraperitoneally and subcutaneously.

As the patient required inotropic support and mechanical ventilation, she was followed up in the intensive care unit. She received packed red blood cells and fresh, frozen plasma. A marked improvement was noticed in her general condition and abnormal laboratory values in the next 24 hours. On the second day, she was moved to a general ward, and intravenous antibiotic therapy was continued. Microbial cultures were shown to contain E. coli and were sensitive to cotrimoxazole and trimethoprim. The patient was discharged from the hospital on the sixth postoperative day. On the follow-up visit, stitches were removed on day 14, the patient was clinically well, and good wound healing was observed.

## Discussion

A tubo-ovarian abscess is a rare yet serious condition associated with high morbidity and potential mortality [2]. The rupture of a tubo-ovarian abscess is a life-threatening emergency and requires prompt surgical intervention. A ruptured tubo-ovarian abscess occurs in approximately 15% of cases. The mortality rate can be up to 4% in cases of rupture, even with modern therapies [3]. Clinical findings suggestive of rupture include acute peritoneal signs, hypotension, tachycardia, tachypnea, or acidosis. The patient presented with all the above clinical findings. However, the presence of anaemia, palpable uterine mass, and unilateral tenderness in the lumbar region posed a challenge to diagnosis. A literature search revealed only one case where a tubo-ovarian abscess ruptured into the anterior abdominal wall [4]. In our patient, the previous laparoscopic myomectomies could have contributed to the spread into the anterior abdominal wall. The development of tubo-ovarian abscess is influenced by several predisposing factors, such as sexual activity, multiple sexual partners, nulliparity, previous episodes of pelvic inflammatory disease, lower socioeconomic status, and the use of intrauterine devices [5]. Our patient had a history of chronic pelvic pain but did not have any of the other associated risk factors mentioned above. The association between tubo-ovarian abscess and endometriosis has also been emphasized in several works of literature. The causative organism is usually polymicrobial with anaerobic predominance. Bacteria move upwards from the lower genital tract, leading to the formation of an inflammatory mass involving the fallopian tube, ovary, and potentially other adjacent pelvic organs. While both transvaginal ultrasound and computed tomography can be used for diagnosis, computed tomography offers higher sensitivity and can differentiate it from gastrointestinal pathologies with similar presentations [6]. The standard treatment for tubo-ovarian abscesses involves broad-spectrum antibiotics, with a success rate of approximately 70%. The majority of small abscesses with diameters less than 7 cm usually resolve with antibiotics [7,8]. Large-size tubo-ovarian abscesses in the Asian population with a high body mass index were identified as independent risk factors for a failed response to medical management [9]. A surgical approach is indicated in cases of rupture or tubo-ovarian abscess persistence. Surgical treatment comprises laparotomy or laparoscopy with drainage of the abscess, unilateral or bilateral salpingectomy, unilateral or bilateral adnexectomy, and hysterectomy. Surgery for a tubo-ovarian abscess can be technically challenging and associated with complications. The risk of bowel injury increases because of its adherence to the septic mass. Other complications reported with surgical management are wound infection, wound dehiscence, vaginal vault hematoma, and persistence of pelvic abscess. Imaging-guided drainage of abscesses paired with antibiotics offers an alternative approach. Minimally invasive procedures for TO abscesses performed by the laparoscopy approach and transvaginal ultrasound-guided drainage are associated with less morbidity and a shorter hospital stay [10-12].

## Conclusions

A spontaneous rupture into the anterior abdominal wall is an uncommon presentation of tubo-ovarian abscess. Early detection and treatment of tubo-ovarian abscesses can prevent complications such as abscess rupture and sepsis. In certain life-threatening situations, aggressive surgery is a justifiable treatment option for tubo-ovarian abscesses.
